# Preferential Processing of Social Features and Their Interplay with Physical Saliency in Complex Naturalistic Scenes

**DOI:** 10.3389/fpsyg.2017.00418

**Published:** 2017-03-30

**Authors:** Albert End, Matthias Gamer

**Affiliations:** ^1^Department of Systems Neuroscience, University Medical Center Hamburg-EppendorfHamburg, Germany; ^2^Department of Psychology, Julius Maximilians University of WürzburgWürzburg, Germany

**Keywords:** social attention, overt attention, physical saliency, visual perception, naturalistic scenes, eye movements, gaze prediction

## Abstract

According to so-called saliency-based attention models, attention during free viewing of visual scenes is particularly allocated to physically salient image regions. In the present study, we assumed that social features in complex naturalistic scenes would be processed preferentially irrespective of their physical saliency. Therefore, we expected worse prediction of gazing behavior by saliency-based attention models when social information is present in the visual field. To test this hypothesis, participants freely viewed color photographs of complex naturalistic social (e.g., including heads, bodies) and non-social (e.g., including landscapes, objects) scenes while their eye movements were recorded. In agreement with our hypothesis, we found that social features (especially heads) were heavily prioritized during visual exploration. Correspondingly, the presence of social information weakened the influence of low-level saliency on gazing behavior. Importantly, this pattern was most pronounced for the earliest fixations indicating automatic attentional processes. These findings were further corroborated by a linear mixed model approach showing that social features (especially heads) add substantially to the prediction of fixations beyond physical saliency. Taken together, the current study indicates gazing behavior for naturalistic scenes to be better predicted by the interplay of social and physically salient features than by low-level saliency alone. These findings strongly challenge the generalizability of saliency-based attention models and demonstrate the importance of considering social influences when investigating the driving factors of human visual attention.

## Introduction

Humans as social beings permanently face diverse forms of social interactions, which in turn require possessing a broad set of social functions. Whereas previous research on social capabilities largely focused on higher order functions (e.g., theory of mind and empathy), the basic processes underlying these functions (e.g., social attention) are explored less extensively. However, social attention is at the heart of every complex social capability because without first allocating attention to other human beings or to aspects in the environment which they attend to, it is essentially impossible to infer their intentions or to feel empathy with them (Adolphs, 2010).

The majority of research on social information processing relied on pictures of either schematic or isolated real faces to study gaze orienting or face perception. Although such studies provided evidence that observers exhibit a bias to direct their attention to the eyes of another person (e.g., Walker-Smith et al., 1977; Pelphrey et al., 2002; Henderson et al., 2005; Gamer and Büchel, 2009) or to locations gazed at by others (e.g., Friesen and Kingstone, 1998; Driver et al., 1999; Langton and Bruce, 1999; Ricciardelli et al., 2002), the use of impoverished stimuli such as faces shown in isolation from their surrounding massively simplifies the challenges of natural human vision. In real life, social elements such as faces, heads, or bodies of others are typically – if at all – only one feature of the visual input among several other aspects (e.g., various kinds of objects) and before being able to process specific details of others’ faces, observers in a first step need to allocate their attention to them (Birmingham and Kingstone, 2009; Birmingham et al., 2009a; Kingstone, 2009). In recent years, researchers started to seriously question the impoverished approach and began to use more complex stimuli such as photographs of naturalistic scenes containing people. In this context, several studies yielded support that humans have a tendency to direct their gaze to other persons (especially their heads and eyes; e.g., Smilek et al., 2006; Birmingham et al., 2007, 2008a,b, 2009a; Castelhano et al., 2007; Fletcher-Watson et al., 2008; Zwickel and Vo, 2010; see also Kano and Tomonaga, 2011). This research is of extraordinary importance for the study of social attention because it laid the foundation for the application of ecologically valid stimuli. However, previous studies are not in every respect unimpeachable. For example, even though previously used stimulus material depicted humans in naturalistic environments, these photos frequently seemed to contain people in relatively large size and, apart from that, only a rather limited amount of complex information which might have had the potential to capture the observer’s attention. Correspondingly, there is a lack of studies which explicitly identified non-social scene aspects that are at least equally conspicuous (e.g., regarding low-level features) than depicted persons and directly compared to what degree people and such other locations receive attention.

In addition, most research on processing social information is not connected sufficiently to prevalent attention theories. Traditional models assume attention to be driven by stimulus characteristics (so-called bottom-up processing) as well as higher order motivational goals (so-called top-down processing; Corbetta and Shulman, 2002; Knudsen, 2007; Corbetta et al., 2008). Moreover, computational approaches within this framework consider attention to be particularly directed to those aspects of the visual field which are physically highly salient (Itti and Koch, 2001). For example, according to the most prominent of these computational models by Itti and Koch (Itti et al., 1998; Itti and Koch, 2000), those locations of a visual input that stand out from the background in terms of their low-level features (i.e., color, intensity, and orientation contrast) are calculated on various spatial scales using biologically plausible center-surround differences. Subsequently, the distributions of conspicuous locations of the different spatial scales and the three low-level features are integrated into a so-called saliency map which is considered to guide the allocation of attention. Following the seminal work of Itti and Koch, a diverse set of computational saliency approaches has been developed (for reviews, see e.g., Judd et al., 2012; Borji and Itti, 2013). Although research provided empirical support for saliency-based attention models (e.g., when humans freely viewed or memorized visual scenes; Parkhurst et al., 2002; Foulsham and Underwood, 2008), there are several recent studies indicating circumstances under which these models work less well or fail completely (e.g., under presence of top–down influences from visual search tasks; Foulsham and Underwood, 2007; Henderson et al., 2007; Einhäuser et al., 2008a; see also e.g., Stirk and Underwood, 2007; Einhäuser et al., 2008b; Santangelo, 2015; Santangelo et al., 2015; but see e.g., Borji et al., 2013; Spotorno et al., 2013). Importantly, prior research also shed light on the power of saliency-based predictions in the context of social information. For example, several studies yielded support that the influence of physical saliency on gazing behavior may be especially weak for socially relevant stimuli (e.g., Nyström and Holmqvist, 2008; Birmingham et al., 2009a,b; Fletcher-Watson et al., 2009; Zwickel and Vo, 2010; Hall et al., 2011; Scheller et al., 2012; Suda and Kitazawa, 2015; see also Kano and Tomonaga, 2011; Solyst and Buffalo, 2014) and a number of modeling approaches provided evidence that further sources of information (e.g., locations of faces) may be considered in addition to low-level saliency (e.g., Cerf et al., 2008, 2009; Marat et al., 2013; Xu et al., 2014; Parks et al., 2015; for a review, see Tatler et al., 2011). However, the attentional influence of physical saliency in the context of social information has so far not been characterized sufficiently. For example, in previous research, stimuli mostly only consisted of isolated social features (e.g., faces, human figures) or visual scenes which did not allow for a definite distinction between the influences of social attention and low-level saliency. Specifically, physical saliency of (parts of) people in these visual scenes was frequently either not reported at all, higher than for the majority of other considered image regions or not compared statistically to those other locations. Moreover, to the best of our knowledge, the power of saliency-based predictions has so far not been compared systematically between scenes including social information and scenes depicting only non-social aspects in human observers (but only in capuchin monkeys; see Berger et al., 2012).

**FIGURE 1:**
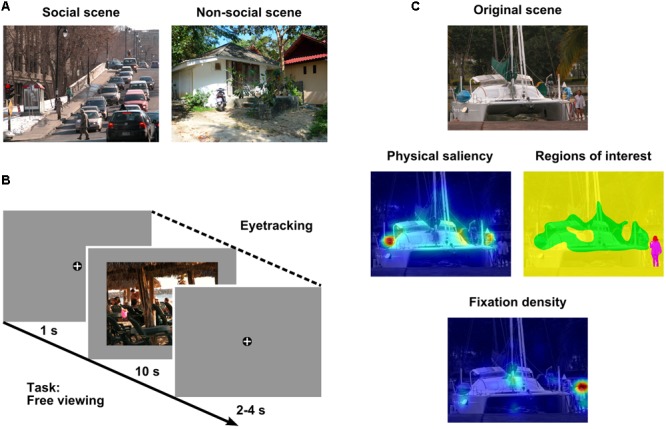
**Methods. (A)** Example of a social (left) and a non-social (right) scene. **(B)** Example trial containing a social scene. Scenes were preceded and followed by a fixation cross. Participants freely viewed each visual scene for 10 s while their eye movements were recorded. Note: Size of fixation cross is not to scale. **(C)** Example original scene (top row), corresponding regions of interest (ROIs: red = head, magenta = body, yellow = area of lower saliency, green = area of higher saliency; middle row, right), and heat maps for physical saliency (middle row, left) and fixation density (bottom row) overlaid on the original scene. Note: Cold colors indicate low saliency or fixation density, warm colors represent high saliency or fixation density. Photographs of visual scenes reproduced by kind permission of A. Marchewka (Marchewka et al., 2013) and F. A. A. Kingdom (Olmos and Kingdom, 2004).

To close these gaps, the present study aimed at characterizing the role of human social attention in the context of popular saliency-based attention models by means of ecologically valid stimuli. One possible explanation for the described evidence that saliency-based attention models may be particularly poor in predicting gazing behavior for socially relevant stimuli could be based on the mentioned findings from research on allocating attention to other people (see also e.g., Nyström and Holmqvist, 2008; Birmingham et al., 2009b). In particular, we hypothesized that social features such as heads or bodies in complex naturalistic scenes would be processed preferentially regardless of their physical saliency. Therefore, we expected saliency-based attention models to lose power in predicting gazing behavior when social elements are present in the visual field. To test this assumption, participants freely viewed color photographs of complex naturalistic social (e.g., including heads, bodies) and non-social (e.g., including landscapes, objects) scenes while their eye movements were recorded (see **Figures 1A,B**). Crucially, we systematically compared the extent to which physical saliency predicted fixations between social and non-social scenes and examined to what degree participants directed their attention to social features or image locations with certain physical saliency. Importantly, explicitly comparing the amount of physical saliency of each of these regions as well as taking care during stimulus selection that social features were not confounded by low-level saliency enabled us to disentangle potential influences of social attention and physical saliency. Interestingly, saliency-based attention models were previously demonstrated to perform best for the very first fixations after stimulus onset (e.g., Parkhurst et al., 2002; but see Tatler et al., 2005) and several studies provided support that a bias of directing gaze to other people may also be present for such early fixations (e.g., Birmingham et al., 2008a, 2009b; Fletcher-Watson et al., 2008). We aimed at distinguishing both influences on early eye movements more comprehensively by carrying out detailed analyses of the first five fixated scene locations for social and non-social scenes. Importantly, specifically examining early, potentially automatic shifts of attention allowed us to more closely inspect the nature of the mechanisms underlying the allocation of attention in naturalistic scenes. Finally, we applied a recently proposed linear mixed model analysis approach (Nuthmann and Einhäuser, 2015) which constitutes an important advancement over previous studies because it enabled us to compare physical saliency and social features as well as their interaction as predictors of fixations within a shared model.

## Materials and Methods

### Participants

Thirty-one volunteers (20 males; mean age: 26.6 years; range: 20–43 years; *SD*: 4.3 years) participated in the study. This sample size was required for detecting medium effects (*d* = 0.50) in paired comparisons (one-tailed) with a power of at least 0.85. All participants gave written informed consent and received monetary compensation. They reported normal or corrected to normal vision and most of them were students of various disciplines (65%). None of the participants reported to have a history of neurological or psychiatric illness or to receive centrally acting medication. The study was approved by the ethics committee of the German Psychological Society (DGPs) and conducted in accordance with the Declaration of Helsinki.

### Stimuli

The stimulus set consisted of 160 color photographs of complex naturalistic scenes which depicted various kinds of indoor (e.g., from living or working places) and outdoor (e.g., from cities or the countryside) scenes including mainly objects and occasionally animals. We considered scenes as social if they included (parts of) a human being (e.g., heads, bodies, or body parts). Half of the scenes contained such social features (“social scenes”) and the other half depicted solely non-social content (i.e., did not include human beings, “non-social scenes”; see **Figure 1A**). Both social and non-social scenes varied in emotional quality from negative to positive (including neutral scenes).

The stimuli were taken from various image databases (Emotional Picture Set [EmoPicS; Wessa et al., 2010]; International Affective Picture System [IAPS; Lang et al., 2008]; McGill Calibrated Colour Image Database [Olmos and Kingdom, 2004]; Nencki Affective Picture System [NAPS; Marchewka et al., 2013]; Object and Semantic Images and Eyetracking dataset [OSIE; Xu et al., 2014]) and the internet (e.g., Google picture search, flickr). The stimuli were required to have sufficient depth of field and complexity (i.e., to have several kinds of visual information in the fore- and background), to be naturalistic (i.e., not drawn or manipulated with artificial photographical effects), and previously unfamiliar to the sample. Scenes containing a lot of conspicuous text were avoided. Moreover, stimuli had to be in sufficiently high resolution and quality to allow for cropping or rescaling to a resolution of 1,200 × 900 pixels. Image editing was performed with the software GIMP (Version 2.8.10, GNU Image Manipulation Program, The GIMP Team).

These selection criteria were applied equally to all scenes to minimize the risk of potential confounds between the two sets of stimuli (i.e., social and non-social scenes). In addition, stimulus comparability was validated using three computational approaches to determine image complexity and clutter according to Rosenholtz et al. (2007). Importantly, two-sample *t*-tests (two-tailed) revealed social and non-social scenes to be similar regarding the suggested measures of feature congestion (*t*[158] = 0.07, *p* = 0.94, *d* = 0.01; social scenes: *M* = 4.18, *SD* = 1.09; non-social scenes: *M* = 4.17, *SD* = 1.04), subband entropy (*t*[158] = 0.67, *p* = 0.50, *d* = 0.11; social scenes: *M* = 3.89, *SD* = 0.33; non-social scenes: *M* = 3.93, *SD* = 0.39), and edge density (using Matlab^®^’s [Mathworks, Inc., Natick, MA, USA] Canny (1986) edge detector; *t*[158] = 0.83, *p* = 0.41, *d* = 0.13; social scenes: *M* = 4.79, *SD* = 2.08; non-social scenes: *M* = 5.08, *SD* = 2.36). Moreover, stimulus comparability was validated by means of image ratings obtained from the participants after the actual experiment. In detail, participants evaluated each image regarding valence and arousal using computerized versions of the 9-point Self-Assessment Manikin scales (SAM; Lang, 1980; Hodes et al., 1985; Bradley and Lang, 1994) as well as with respect to personal relevance on a self-constructed scale following the same principle as SAM. Importantly, two-sample *t*-tests (two-tailed) on the average picture ratings across participants showed social and non-social scenes to be similar regarding valence (*t*[158] = 0.52, *p* = .60, *d* = 0.08; social scenes: *M* = 4.78, *SD* = 1.76; non-social scenes: *M* = 4.94, *SD* = 2.04), arousal (*t*[158] = 1.41, *p* = 0.16, *d* = 0.22; social scenes: *M* = 4.51, *SD* = 1.33; non-social scenes: *M* = 4.18, *SD* = 1.63), and personal relevance (*t*[158] = 1.25, *p* = 0.21, *d* = 0.20; social scenes: *M* = 3.84, *SD* = 0.81; non-social scenes: *M* = 4.01, *SD* = 0.91). Thus, potential differences in attentional effects between scenes including human beings and scenes depicting solely non-social content would neither be due to confounds in image complexity nor due to confounds in emotional quality.

### Apparatus

The software Presentation^®^ 17.0 (Neurobehavioral Systems, Inc., Berkeley, CA, USA) was used to control stimulus presentation and data recording. Each stimulus was presented centrally in front of a gray background on a 20.1″ LCD screen with a refresh rate of 60 Hz. The size of each stimulus was 30.6 × 23.0 cm corresponding to 32.5° × 24.7° of visual angle at the fixed viewing distance of 52.5 cm.

Eye movements were recorded at constant lighting conditions from the right eye at a sampling rate of 1,000 Hz using a video-based eye tracker (EyeLink 1000, SR Research, Ottawa, ON, Canada) with a spatial resolution of 0.01° and a spatial accuracy of 0.5°. Additionally, physiological responses (electrocardiography [ECG], respiration, skin conductance [SCR], electromyography [EMG] of zygomaticus major) were measured with a Biopac MP 100 (Biopac Systems, Inc.) device but they are not part of this manuscript.

### Procedure

All subjects participated in the study individually. They were informed about the general procedure of the study and completed the informed consent form as well as a questionnaire asking for sociodemographic data and other aspects relevant to the study (e.g., age, sex, defective vision). Subsequently, the measurement instruments (see Section Apparatus) were attached and participants were given a detailed verbal explanation of the experimental task. In addition, they were asked to avoid large head and body movements and to keep their heads in a head rest ensuring a fixed viewing distance of 52.5 cm during the experimental blocks. Before the beginning of the experiment, the eye tracking system was calibrated using a nine-point grid and the calibration was validated.

The experiment started with 6 training trials containing scenes which were not part of the actual experiment but followed the same principle (i.e., 50% social and 50% non-social scenes). These training trials were followed by 160 experimental trials containing 80 social and 80 non-social scenes in randomized order. In each trial, participants were presented with a fixation cross for 1 s followed by a visual scene for 10 s followed by a fixation cross for a random period of time between 2 and 4 s. The task of the participants was to fixate the fixation cross continuously when it was present and to freely view each visual scene (e.g., as if they were looking at photographs in a newspaper or magazine; see **Figure 1B**). Breaks were allowed after every 42 trials and were followed by a new calibration (including a validation) of the eye tracking system.

After the eye tracking experiment, the measurement instruments were detached and the participants performed ratings of the previously presented scenes and completed several psychometric tests and questionnaires. These rating, test, and questionnaire data will be pooled across several studies and are not part of this manuscript.

### Data Processing and Analysis

Data processing and analysis was performed with the open-source statistical programming language R^1^ and Matlab^®^ R2014a (Mathworks, Inc., Natick, MA, USA). The a priori significance level of α = 0.05 was used for all analyses. As effect size estimates, we report Cohen’s *d* (Cohen, 1988) for two-sample *t*-tests, Cohen’s *d* for paired data (Dunlap et al., 1996; Cohen, 1988; Morris and DeShon, 2002) for paired *t*-tests, and $\eta_{p}^{2}$ for analyses of variance (ANOVAs). Huynh-Feldt’s 𝜀 is reported for all repeated-measures ANOVAs containing more than one degree of freedom in the numerator to account for potential violations of the sphericity assumption.

First, a saliency map (1,200 × 900 pixels) according to the Graph-Based Visual Saliency (GBVS) model (Harel et al., 2007) was calculated for each visual scene (see **Figure 1C**). The GBVS model was implemented in the current study because it was previously demonstrated to be one of the best performing saliency-based attention models which is biologically plausible, available with Matlab^®^ source code, and applicable without initial machine learning of feature weights by means of ground truth training data (Harel et al., 2007; Judd et al., 2012; Borji and Itti, 2013; see also the MIT Saliency Benchmark website of Bylinskii et al., 2016)^2^. In GBVS, those locations of a visual input that are most different from their surrounding in terms of their low-level features (i.e., color, intensity, and orientation) are calculated on various spatial scales using graph-based dissimilarity representations which are interpreted as Markov chains. Subsequently, the distributions of conspicuous locations of the different spatial scales and the three low-level features are integrated into an overall saliency map. The values of the saliency maps range from 0 to 1. Importantly, two-sample *t*-tests (two-tailed) revealed that the saliency maps of our social and non-social scenes had comparable mean values (*t*[158] = 0.97, *p* = 0.34, *d* = 0.15; average mean for social scenes: 0.26; average mean for non-social scenes: 0.26) and standard deviations (*t*[158] = 1.53, *p* = 0.13, *d* = 0.24; average *SD* for social scenes: 0.20; average *SD* for non-social scenes: 0.21).

Second, the standard configuration of SR Research’s EyeLink DataViewer software was used to parse eye movements into saccades and fixations. Hence, saccades were identified as eye movements exceeding a velocity threshold of 30°/sec or an acceleration threshold of 8,000°/sec^2^ and fixations were identified as time periods between saccades. These thresholds are common in eye-tracking research (Holmqvist et al., 2011, pp. 150ff.) and were successfully used in a number of previous studies of our group (e.g., Scheller et al., 2012; Boll and Gamer, 2014; Boll et al., 2016). We decided to use constant thresholds across all participants as compared to individualized data scoring to ensure objectivity of data processing. For each trial, fixations which occurred during scene presentation and started after scene onset were considered for data processing and analysis. Next, for each participant, x and y coordinates of fixations for each scene were drift corrected with reference to the mean position data of fixations during a 300 ms baseline time interval directly before the onset of the respective scene (i.e., when the central cross was fixated). In order to avoid the use of distorted position data of baseline fixations (i.e., when participants did not fixate the central cross) for drift correction, a recursive outlier removal was conducted using each participant’s distribution of baseline position data of all scenes. In detail, separately for x and y coordinates, the lowest and the highest values of baseline position data were removed from the respective distribution and it was checked whether any of these two extreme values was located more than three standard deviations below or above the mean of the remaining distribution. If this was the case, the extreme value was discarded permanently and the algorithm was recursively applied to the lowest and highest values of the remaining distribution. This process was performed until there were no values left which met the removal criterion. Subsequently, the baseline position data of all scenes including a removed x or y baseline coordinate or missing baseline data (number of social scene trials: *M* = 3.48, *SD* = 3.84; number of non-social scene trials: *M* = 4.06, *SD* = 4.35) were replaced by the means of all scenes with valid baseline position data. After fixations were drift corrected, they were used to create fixation density maps (see **Figure 1C**). For each participant and scene, an empty two-dimensional map (1,200 × 900 pixels) was generated, the respective fixations were weighted by their fixation durations in milliseconds (average fixation durations: *M* = 276.9 ms, *SD* = 47.8 ms for social scenes and *M* = 280.6 ms, *SD* = 45.4 ms for non-social scenes), and these weighted fixation values were additively assigned to the map at the pixel position of the fixation. Afterward, the resulting map was smoothed with a two-dimensional isotropic Gaussian kernel with a standard deviation of 36 pixels corresponding to 1° of visual angle using the R package spatstat (version 1.38-1; Baddeley and Turner, 2005). Therefore, two standard deviations (one in positive and one in negative direction) of the kernel amounted to 2° of visual angle roughly resembling the functional field of the human fovea centralis. Finally, the scale of the fixation density map was normalized to range from 0 to 1.

Third, for each participant and scene, the fixation density map was compared to the saliency map using three different metrics (Wilming et al., 2011). These metrics measured the divergence of the distributions of physical saliency and fixation density (Kullback–Leibler divergence, *D_KL_*, Kullback, 1959) and the saliency-based prediction of fixation density (area under the receiver-operating-characteristics curve, *AUC*, Fawcett, 2006; Pearson product-moment correlation coefficient, *r*). For calculating these metrics, both saliency and fixation density maps were rescaled such that their values aggregated to 1 each. In addition, exclusively for *AUC*, fixation density maps were binarized by assigning a value of 1 if the fixation density value was higher than this map’s mean fixation density value and 0 otherwise. Subsequently, for each participant, the mean of each of the three metrics for comparing saliency and fixation density maps was calculated separately for social and non-social scenes. Finally, a paired *t*-test (two-tailed) was performed for each of the three metrics to compare the participants’ means for social and non-social scenes (*n* = 31). In this and all following analyses involving eye movement data, trials were only considered as valid if the aggregated time of blink occurrences (defined by the EyeLink system as periods of missing pupil data) during scene presentation was shorter than 2 s. This applied on average to 78.9 (*SD* = 2.0) social scene trials and to 78.7 (*SD* = 2.9) non-social scene trials.

Fourth, the pixel coordinates of the following four regions of interest (ROIs) were defined for each social scene: heads, bodies (excluding heads and including torso, arms, hands, legs, feet) as well as further areas of lower and higher physical saliency (see **Figure 1C**). On average, 2.15% (*SD* = 2.46%) of a scene were covered by heads, 8.95% (*SD* = 8.23%) by bodies, 71.35% (*SD* = 8.26%) by areas of lower saliency, and 17.56% (*SD* = 2.07%) by areas of higher saliency. The two first-mentioned ROIs were determined by drawing them manually using GIMP. In order to define the two last-mentioned ROIs, the social scene’s saliency map was considered for those image regions which had not already been assigned to the head or body ROI. Saliency values smaller or equal to the eighths saliency decile were defined as area of lower saliency and the remaining scene part as area of higher saliency. Whereas the selection of the eighths saliency decile as the criterion for defining these two ROIs was arbitrary, this cut-off fulfilled the purpose of enabling us to identify image regions which contained no social features (i.e., heads or bodies) but had particularly high physical saliency. Importantly, the identification of such image regions constituted an essential prerequisite for being able to disentangle potential effects of attentional allocation to social versus physically salient information. Next, for each social scene (*n* = 80), the defined ROIs were used in combination with the saliency map to specify the relative amount of physical saliency of each ROI by dividing the mean saliency per ROI by the mean saliency of the whole scene. Subsequently, a one-way ANOVA with repeated measurements on the factor ROI (head, body, area of lower saliency, area of higher saliency) was performed on these data. It has to be mentioned that care was taken at the stage of stimulus selection such that social features were not particularly often image regions with highest physical saliency. This was another necessary prerequisite for being able to disentangle potential effects of social attention and physical saliency. In addition, for each participant and social scene, the defined ROIs and the fixation density map were used to determine the relative extent to which each ROI was fixated. In detail, the sum of fixation density values was calculated for each ROI and then divided by the sum of fixation density values for the whole scene. Afterward, this proportion score was normalized by dividing it by the area of the respective ROI to control for the issue that the probability of receiving more fixations is higher for larger than smaller areas (Smilek et al., 2006; Birmingham et al., 2009a). Finally, for each participant (*n* = 31), the mean of this relative area-normalized sum of fixation density was calculated for each ROI across all social scenes and a one-way ANOVA with repeated measurements on the factor ROI (head, body, area of lower saliency, area of higher saliency) was performed on these data. It is worth mentioning that care was taken at the stage of stimulus selection such that social features were not particularly often located very close to scene center because previous research provided evidence that humans typically exhibit a central fixation bias, i.e., a tendency to fixate the center of images more than the periphery when viewing them on a screen (e.g., Mannan et al., 1996; Tatler et al., 2005; Tatler, 2007; Tseng et al., 2009; Vincent et al., 2009). Thus, ensuring that social features were not systematically located closer to scene center than areas of higher saliency was a necessary prerequisite for avoiding potential effects of social attention (versus physical saliency) to be confounded with general central fixation tendencies. Importantly, the average of the mean distance of social features to scene center (*M* = 9.60°, *SD* = 3.41° for heads; *M* = 9.71°, *SD* = 3.16° for bodies) lay between the corresponding distance to scene center values of areas of higher saliency (*M* = 7.13°, *SD* = 0.84°) and areas of lower saliency (*M* = 12.47°, *SD* = 0.53°).

Fifth, for each participant and scene, the drift corrected fixations, which were already used to create fixation density maps, were considered in combination with the saliency map to determine the relative amount of physical saliency at each of the first five fixated scene locations (see Freeth et al., 2011). Because participants made on average 31.7 (*SD* = 4.0) fixations when viewing social scenes and 31.1 (*SD* = 4.0) when perceiving non-social scenes, the exploration of the first five fixations aims particularly at investigating the influence of physical saliency on very early eye movements. The relative amount of saliency at each of the first five fixated locations was specified by dividing the mean saliency of a circular area with a diameter of 2° of visual angle (resembling the functional field of the human fovea centralis) around the position of the respective fixation by the mean saliency of the whole scene. Next, for each participant (*n* = 31), the average of this relative mean saliency was calculated for each of the first five fixated locations, separately for social and non-social scenes. Subsequently, a two-way ANOVA with repeated measurements on the factors fixation number (1, 2, 3, 4, 5) and scene content (social, non-social) was calculated on these data. In addition, for each participant (*n* = 31) and social scene, the mentioned drift corrected fixations and the defined ROIs were used to specify for each of the first five fixations which ROI was looked at. Afterward, for each participant and for each of the first five fixations, the relative frequency that each ROI was fixated across all social scenes was determined by dividing the frequency that each ROI was fixated by the frequency that any ROI was fixated. Furthermore, each relative frequency score was normalized by dividing it by the mean area of the respective ROI across all social scenes whose data were represented in the respective relative frequency score. This normalization was applied to control for the fact that the probability of receiving a fixation is higher for larger than smaller areas (Smilek et al., 2006; Birmingham et al., 2009a). Finally, a two-way ANOVA with repeated measurements on the factors fixation number (1, 2, 3, 4, 5) and ROI (head, body, area of lower saliency, area of higher saliency) was performed on these data. It has to be mentioned that trials of which baseline position data were replaced by mean baseline position data for drift correction were excluded from both described analyses involving the exploration of the first five fixations because the probability is very high that participants did not fixate the central cross directly before the onset of the scene in these trials. Therefore, the starting position to view a scene most probably differs systematically between these trials and trials in which the instruction to fixate the central cross before scene presentation was adhered. Whereas such a difference in starting positions does not necessarily constitute a problem for the creation of fixation density maps which comprise fixations for the whole scene viewing duration, it could especially influence the locations of fixations occurring very early after the onset of a scene which would particularly bias the described analyses involving the exploration of the first five fixations.

Finally, a recently proposed analysis approach which combines a scene patch analysis with linear mixed models (Nuthmann and Einhäuser, 2015) was used to further compare the power of physical saliency and social features in predicting gazing behavior. This approach constitutes an important improvement beyond the previously described data analyses because it enabled us to incorporate physical saliency and social features as well as their interaction as predictors of fixations in a shared model. Moreover, it facilitated the consideration of trial specific data characteristics because it did not require aggregation of participants and experimental conditions across trials. In detail, each social scene (1,200 × 900 pixels) was overlaid with a 12 × 9 grid to divide it into 108 quadratic scene patches of 100 × 100 pixels each. Subsequently, for each participant and social scene, the fixation density map was used to calculate the mean fixation density of each of these quadratic scene patches. This mean fixation density of each scene patch was further divided by the average mean fixation density across all patches of a scene to get a relative index of the extent that a participant looked at each scene patch. Accordingly, for each social scene, the relative mean saliency of each scene patch was specified by means of the saliency map. Furthermore, each social scene’s ROIs were used to determine the relative amount that the area of each scene patch was covered by social features. In detail, the relative area covered by one or more heads was calculated for each scene patch and then divided by the average relative area covered by one or more heads across all patches of a scene. Correspondingly, the relative area covered by one or more bodies was specified for each social scene’s patches. In addition, in the context of this analysis approach, it is worth reconsidering the mentioned empirical evidence that humans typically exhibit a central fixation bias when viewing images on a screen (e.g., Mannan et al., 1996; Tatler et al., 2005; Tatler, 2007; Tseng et al., 2009; Vincent et al., 2009). Although the implemented GBVS saliency model already implicitly incorporates a central bias due to the used graph-based dissimilarity representations (Harel et al., 2007), we followed Nuthmann and Einhäuser (2015) by also explicitly including the central bias, operationalized as the Euclidean distance between the center of each grid cell and the scene center, in our model analyses. Importantly, this enabled us to compare physical saliency, social features, and their interaction as predictors of fixations largely isolated from this general central viewing tendency. Thus, the scene patch data (*n* = 264,276) of all participants and social scenes (31 participants; *M* = 78.93548 social trials fulfilling the blink criteria described above; 108 scene patches) were used to determine the power of the four features (i) distance from scene center, (ii) relative mean saliency, (iii) relative amount of heads, and (iv) relative amount of bodies of a scene patch in predicting the relative mean fixation density of this scene patch (i.e., the extent to which participants looked at it) by means of linear mixed models. These models were implemented using the lme4 package (version 1.1-7; Bates et al., 2014) for R with the bobyqa optimizer. Model estimates were chosen to optimize the restricted maximum likelihood (REML) criterion and the predictor’s *p*-values were obtained based on Satterthwaite’s approximation of degrees of freedom using the lmerTest package (version 2.0-25; Kuznetsova et al., 2015). The predictors were transformed to have mean 0 and standard deviation 1. Importantly, the notation “mixed” in linear mixed models refers to the fact that these models contain both fixed and random effects. The following models with different fixed effects were tested: We built models including one of the four predictors exclusively (models 1a, 1b, 1c, 1d), a model containing both distance from scene center and relative mean saliency (model 2), models incorporating distance from scene center, relative mean saliency and either relative amount of heads or relative amount of bodies (models 3a, 3b), and finally a model containing all four predictors simultaneously (model 4). The model which included all predictors at once was tested both with and without interaction terms of relative mean saliency and relative amount of heads or bodies (models 5a, 5b, 6).

Additionally, models were compared incrementally using likelihood ratio tests. Therefore, all models were required to have the same random effects structure which was specified as including participant ID and scene ID as random intercepts. For model comparison, model estimates were chosen to optimize the maximum likelihood (ML) criterion instead of the REML criterion. Furthermore, as the calculation of an index of goodness of model fit is nontrivial in the context of linear mixed models, an analog of the coefficient of determination (*R^2^*) was calculated for each model using the square number of the correlation between the observed data and the values predicted by the model (Cameron and Windmeijer, 1996; Byrnes and Stachowicz, 2009). In addition, the Akaike information criterion (AIC; Akaike, 1974) was calculated (using the ML fitted models) because this index of goodness of model fit also considers the principle of model parsimony by penalizing models incorporating a larger number of parameters.

Moreover, for a detailed visual inspection of the relationship between the relative mean fixation density of a scene patch and its distance from scene center, relative mean saliency, and relative amount of heads or bodies, the scene patch data of all participants and social scenes were used to determine the deciles of each of the four predictors. For each of these four variables, each scene patch was assigned to its corresponding decile category and the average of relative mean fixation density was calculated for each of these decile bins. Because this data handling groups the scene patches having the 10% lowest or the 10% highest predictor values (or any 10% category in between) together, it enabled us to visually inspect how the average of relative mean fixation density was influenced by the decile bin containing lower or higher predictor values (see **Figure 5**). Two things have to be mentioned: First, because most of the scene patches did not contain any parts of heads or bodies, before applying the decile categorization to the relative amount of heads and the relative amount of bodies of a scene patch, all scene patches depicting no parts of the respective social feature (either heads or bodies) were grouped into an additional bin and the decile categorization described above was applied to all remaining scene patches (i.e., scene patches containing at least minimal parts of the respective social feature). Second, because of the way the distance from scene center predictor was specified, it had only 27 distinct values appearing with different frequencies. Therefore, the decile bins regarding distance from scene center could not contain 10% of the scene patches each (but between about 7 and 13%).

## Results

### Saliency-Based Prediction of Fixations

**FIGURE 2:**
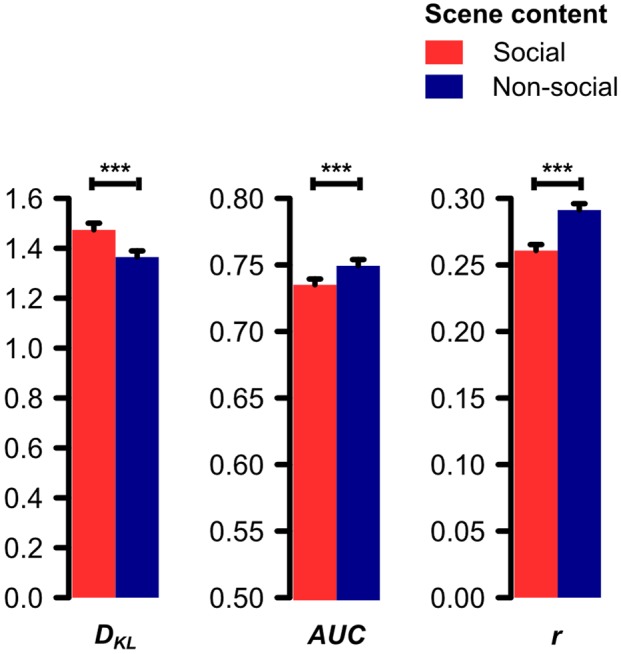
**Divergence (Kullback–Leibler divergence, *D_KL_*) and correspondence (area under the ROC curve, *AUC;* Pearson product-moment correlation coefficient, *r*) between saliency and fixation density maps for social and non-social scenes.** Physical saliency and fixations were found to diverge significantly stronger for social as compared to non-social scenes (*D_KL_*). Correspondingly, physical saliency predicted fixations significantly worse for social as compared to non-social scenes (*AUC* and *r*). Note: *AUC*’s point of origin is set to 0.5 because this value corresponds to random guessing (Fawcett, 2006). Error bars denote *SEM*. ^∗∗∗^*p* < 0.001.

The paired *t*-test on the Kullback–Leibler divergence (*D_KL_*) of the distributions of physical saliency and fixation density for social and non-social scenes revealed saliency and fixations to diverge significantly stronger for social than non-social scenes (*t*[30] = 9.11, *p* < 0.001, *d* = 1.64). Consistently, the paired *t*-tests on the area under the receiver-operating-characteristics curve (*AUC*) and the Pearson product-moment correlation coefficient (*r*) of saliency and fixation density maps revealed the saliency-based prediction of fixations to be significantly worse for social than non-social scenes (*AUC*: *t*[30] = 9.01, *p* < 0.001, *d* = 1.62; *r*: *t*[30] = 10.04, *p* < 0.001, *d* = 1.80; see **Figure 2**).

### Regions of Interest Analysis

**FIGURE 3:**
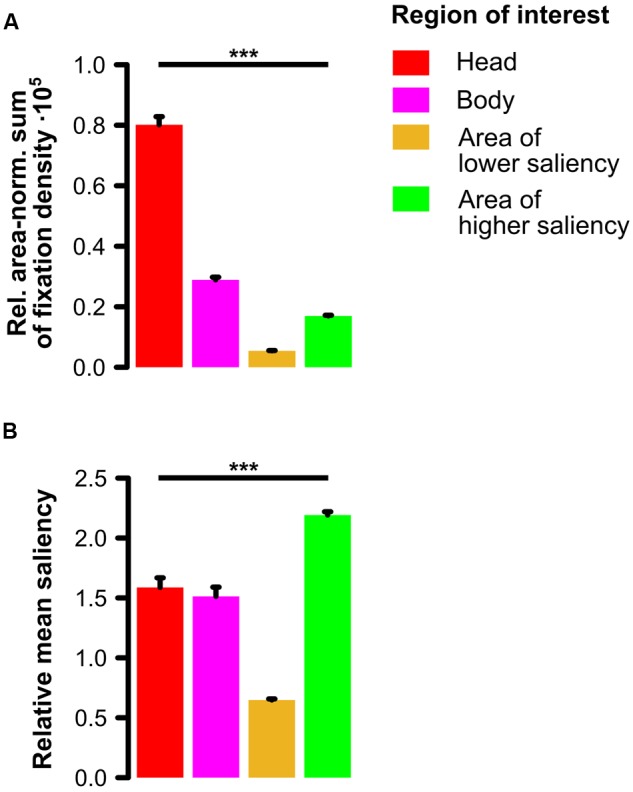
**Comparison of four regions of interest in social scenes. (A)** Relative area-normalized extent to which participants looked at each region of interest (ROI). Social features (especially heads but also bodies) were fixated significantly more than other image regions of lower and higher physical saliency. **(B)** Relative amount of physical saliency of each ROI. Social features constituted areas of intermediate saliency meaning that areas of higher saliency were significantly more salient than heads and bodies which were in turn significantly more salient than areas of lower saliency. Note: Error bars denote *SEM*. ^∗∗∗^*p* < 0.001.

In the one-way ANOVA on the relative area-normalized sum of fixation density values with repeated measurements on the factor region of interest (ROI; head, body, area of lower saliency, area of higher saliency) for social scenes, we observed a significant main effect of ROI (*F*[3,90] = 570.63, 𝜀 = 0.41, *p* < 0.001, $\eta_{p}^{2}$ = 0.95) indicating that heads were looked at the most, followed by bodies which were fixated more than areas of higher saliency which were in turn looked at more than areas of lower saliency (see **Figure 3A**). Paired post hoc *t*-tests (two-tailed) with a Bonferroni-corrected significance level (α = 0.05/6 = 0.0083) revealed all possible pairwise comparisons to be significant (all *t*[30] > 13, all *p <* 10^-13^).

The one-way ANOVA on the relative mean saliency with repeated measurements on the factor ROI (head, body, area of lower saliency, area of higher saliency) for social scenes revealed a significant main effect of ROI (*F*[3,237] = 153.91, 𝜀 = 0.63, *p* < 0.001, $\eta_{p}^{2}$ = 0.66). This main effect indicates that areas of higher saliency were physically more salient than the social features heads and bodies which were in turn more salient than areas of lower saliency (see **Figure 3B**). Paired post hoc *t*-tests (two-tailed) with a Bonferroni-corrected significance level (α = 0.05/6 = 0.0083) revealed that except for the contrast between heads and bodies (*t*[79] = 1.21, *p* = 0.23), all other possible pairwise comparisons (all *t*[79] > 7, all *p <* 10^-10^) were significant.

### Time Course Analysis

**FIGURE 4:**
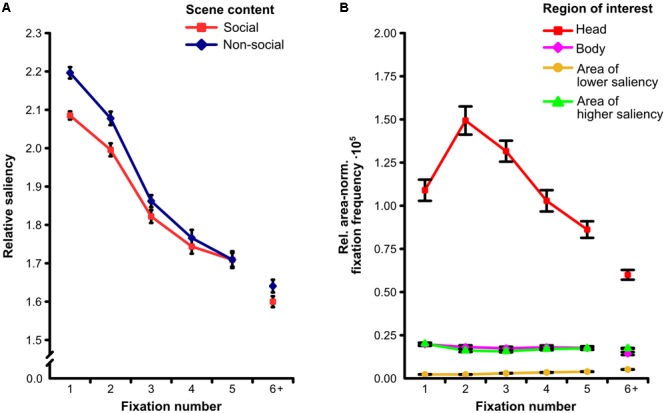
**Fixation time course. (A)** Relative amount of physical saliency at each of the first five fixated scene locations for social and non-social scenes. The lower correspondence between saliency and fixations for social as compared to non-social scenes was significantly more pronounced for the earliest fixations. **(B)** Relative area-normalized frequency that each of four regions of interest (ROIs) was fixated for each of the first five fixations on social scenes. The preferential fixation of heads was significantly more pronounced for the earliest fixations. Note: “6+” indicates the mean value of the sixth to the last fixation. Error bars denote *SEM*.

The two-way ANOVA on the relative mean saliency with repeated measurements on the factors fixation number (1, 2, 3, 4, 5) and scene content (social, non-social) revealed a significant main effect of scene content (*F*[1,30] = 23.32, *p* < 0.001, $\eta_{p}^{2}$ = 0.43) indicating relative mean saliency of fixated locations to be lower for social than for non-social scenes. Moreover, we found a significant main effect of fixation number (*F*[4,120] = 308.16, 𝜀 = 0.81, *p* < 0.001, $\eta_{p}^{2}$ = 0.91). Accordingly, relative mean saliency of fixated locations was highest for the first fixation on a scene and decreased progressively from fixation to fixation. In addition, we observed a significant interaction of fixation number and scene content (*F*[4,120] = 5.44, 𝜀 = 0.86, *p* < 0.001, $\eta_{p}^{2}$ = 0.16). This interaction indicated that the lower relative mean saliency of fixated locations for social as compared to non-social scenes was most pronounced for the first fixation on a scene and declined gradually from fixation to fixation (see **Figure 4A**).

The two-way ANOVA on the relative area-normalized fixation frequency with repeated measurements on the factors fixation number (1, 2, 3, 4, 5) and ROI (head, body, area of lower saliency, area of higher saliency) for social scenes revealed a significant main effect of fixation number (*F*[4,120] = 38.96, 𝜀 = 0.76, *p* < 0.001, $\eta_{p}^{2}$ = 0.57) representing the average of the relative area-normalized fixation frequency across ROIs to be different between the first five fixated locations. Additionally, we found a main effect of ROI (*F*[3,90] = 388.27, 𝜀 = 0.35, *p* < 0.001, $\eta_{p}^{2}$ = 0.93) indicating heads to be looked at more than bodies and areas of higher saliency which were fixated more than areas of lower saliency. Moreover, a significant interaction of fixation number and ROI was observed (*F*[12,360] = 29.43, 𝜀 = 0.27, *p* < 0.001, $\eta_{p}^{2}$ = 0.50). This interaction indicates that the preferential fixation of heads was most pronounced for the earliest fixations on a scene, had its maximum at the second fixation, and gradually declined afterward (see **Figure 4B**).

### Linear Mixed Model Analysis

The β and *p-*values of the linear mixed model analysis comparing the power of the four features distance from scene center, relative mean saliency, relative amount of heads, and relative amount of bodies of a scene patch in predicting the relative mean fixation density on this scene patch are given in **Table 1**. This analysis revealed distance from scene center, relative mean saliency, relative amount of heads, and relative amount of bodies to be significant predictors of relative mean fixation density in models including one of the four features exclusively (models 1a, 1b, 1c, 1d), in a model containing both distance from scene center and relative mean saliency (model 2), in models including distance from scene center, relative mean saliency, and either relative amount of heads or relative amount of bodies (models 3a, 3b), and in a model including all four features simultaneously (model 4). Moreover, if interaction terms of relative mean saliency and relative amount of heads as well as relative mean saliency and relative amount of bodies were added to the model containing all four features concurrently, both interaction terms were observed to be further significant predictors of relative mean fixation density (models 5a, 5b, 6).

**Table 1:** Parameters of linear mixed models predicting the relative mean fixation density on the patches of a social scene by their distance from scene center, relative mean saliency, relative amount of heads, and relative amount of bodies.

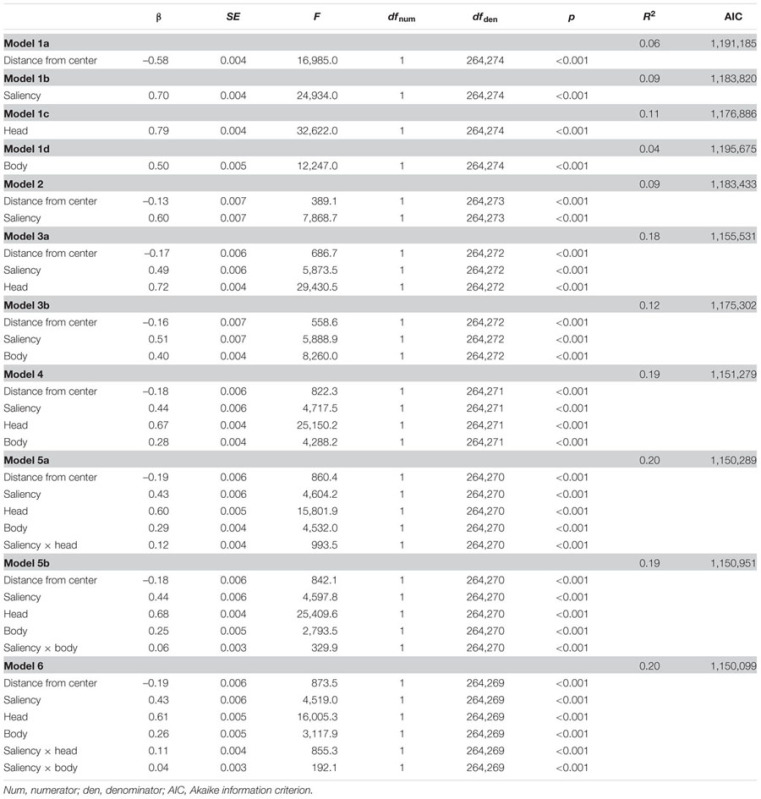

Comparing models incrementally using likelihood ratio tests revealed that a model containing distance from scene center exclusively (model 1a) fitted the data better than a model containing no fixed effects but the same random effects (χ^2^[1] = 16,462.00, *p* < 0.001). Furthermore, a model including distance from scene center and relative mean saliency (model 2) performed better than a model incorporating only distance from scene center (model 1a; χ^2^[1] = 7,753.90, *p* < 0.001) and a model containing distance from scene center, relative mean saliency, and relative amount of heads (model 3a) fitted the data better than a model including only distance from scene center and relative mean saliency (model 2; χ^2^[1] = 27,905.00, *p* < 0.001). Moreover, a model incorporating all four features concurrently (model 4) outperformed a model containing only distance from scene center, relative mean saliency, and relative amount of heads (model 3a; χ^2^[1] = 4,253.80, *p* < 0.001). In addition, a model including all four features and an interaction term of relative mean saliency and relative amount of heads (model 5a) resulted in a higher goodness of fit than a model containing all four features but not the interaction term (model 4; χ^2^[1] = 991.63, *p* < 0.001). Finally, a model incorporating all four features and interaction terms of relative mean saliency and relative amount of heads as well as relative mean saliency and relative amount of bodies (model 6) performed better than a model including all four features and only the interaction term of relative mean saliency and relative amount of heads (model 5a; χ^2^[1] = 192.00, *p* < 0.001). Additionally, model comparisons based on the AIC, which indicates higher goodness of model fit by smaller AIC values, revealed the same pattern of results as the likelihood ratio tests (for the models’ AIC values, see **Table 1**).

**FIGURE 5:**
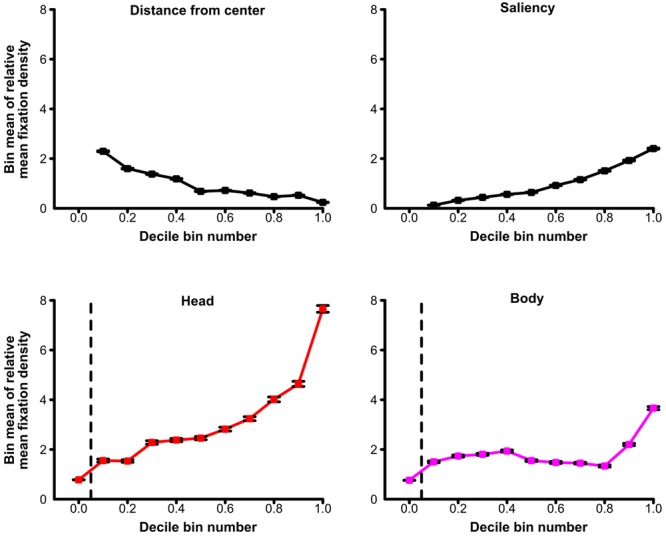
**Predictors of fixations for social scenes.** Average relative fixation density of decile bins containing scene patches with the 10% lowest or the 10% highest values (or any 10% category in between) of the distance from scene center, the relative amount of physical saliency, the relative amount of heads, or the relative amount of bodies in a scene patch. Greater distance from scene center and higher relative amounts of saliency, heads, or bodies of a scene patch are indicated by higher values on the x-axes. A scene patch was fixated more when it contained heads to a higher degree but also when it was physically more salient, when it depicted bodies to a larger extent, and when it was more central in the scene. Note: For the relative amount of heads and the relative amount of bodies of a scene patch, all scene patches which did not contain any parts of the respective social feature (either heads or bodies) are depicted in a separate bin left of the vertical dashed line. The decile bins of the remaining scene patches are illustrated right to the vertical dashed line. The decile bins regarding distance from scene center did not contain 10% of the scene patches each (but between about 7 and 13%; for details, see Section “Materials and Methods”). Error bars denote *SEM*.

For an additional visualization of the relationship between relative mean fixation density and its predictors, see **Figure 5**. For each of the four predictors, this figure depicts the average relative mean fixation density of decile bins containing scene patches with the 10% lowest or the 10% highest predictor values (or any 10% category in between). This allowed the visual inspection of how average relative mean fixation density was influenced by the decile bin containing lower or higher predictor values. Thereby, **Figure 5** further illustrates that the relative mean fixation density of a scene patch increased when this scene patch contained a higher relative amount of heads but also when it had higher relative mean saliency, when it contained a higher relative amount of bodies, and when it had a shorter distance from scene center.

## Discussion

In the present study, we measured eye movements of participants freely viewing color photographs of complex naturalistic social and non-social scenes to examine the role of human social attention in the context of saliency-based attention models. Consistent with our hypothesis, social features (especially heads) were heavily prioritized during visual exploration. Correspondingly, saliency-based attention models lost power in predicting gazing behavior when social elements were present in a visual scene. Importantly, this pattern was most pronounced for the very first fixations. Finally, a linear mixed model approach revealed social features, physical saliency, their interaction, and a central bias to be significant predictors of gazing behavior within a shared model.

The current finding that observers exhibited a strong attentional preference for social features (especially heads) in complex naturalistic scenes is in line with previous studies yielding support that humans have a tendency to direct their gaze to other persons (e.g., Smilek et al., 2006; Birmingham et al., 2007, 2008a,b, 2009a; Castelhano et al., 2007; Fletcher-Watson et al., 2008; Zwickel and Vo, 2010). However, contrary to prior research, we explicitly identified image locations which were physically more salient than depicted people and directly compared to which extent social features and such other regions received attention. Importantly, this enabled us to demonstrate explicitly that gaze is allocated preferentially to other persons even if more conspicuous non-social aspects are present in a visual scene. After impoverished stimuli such as isolated faces were extensively used in previous studies on social perception, in recent years, researchers began to apply ecologically more valid stimulus material (e.g., Smilek et al., 2006; Birmingham et al., 2007, 2008a,b, 2009a; Castelhano et al., 2007; Fletcher-Watson et al., 2008; Zwickel and Vo, 2010). The present findings contribute to this emerging approach because, in real life, other people are typically not the only conspicuous aspect of the visual field (see also Birmingham and Kingstone, 2009; Birmingham et al., 2009a; Kingstone, 2009).

In addition, the current study provides strong evidence that the marked preference for social features cannot be fully explained by physical saliency. First, we took care during stimulus selection that social information was not confounded by low-level saliency. Second, an explicit comparison of the physical saliency of different image regions confirmed social elements to be image regions with only intermediate low-level saliency. Importantly, the majority of previous studies investigating saliency-based predictions in the context of visual scenes containing social information (e.g., Birmingham et al., 2009a,b; Fletcher-Watson et al., 2009; Suda and Kitazawa, 2015) neglected to consider the two mentioned aspects sufficiently. Thus, the current study extends prior reports by providing a clear distinction between the influences of social attention and physical saliency on gazing behavior for complex naturalistic scenes. Moreover, to the best of our knowledge, the present study is the first that systematically compared the extent to which saliency-based attention models predict fixations between scenes including social features and scenes depicting solely non-social content in human observers. In agreement with studies yielding support that the influence of physical saliency on gazing behavior may be especially weak for socially relevant stimuli (e.g., Nyström and Holmqvist, 2008; Birmingham et al., 2009a,b; Fletcher-Watson et al., 2009; Zwickel and Vo, 2010; Hall et al., 2011; Scheller et al., 2012; Suda and Kitazawa, 2015), this comparison allowed us to demonstrate explicitly that the power of saliency-based predictions is considerably reduced when social features are present in a complex naturalistic visual input. Previously, several studies indicated circumstances under which saliency-based attention models work less well or fail completely, for example under presence of top–down influences from visual search tasks (Foulsham and Underwood, 2007; Henderson et al., 2007; Einhäuser et al., 2008a). The current study contributes to this literature by substantiating the notion that physical saliency is insufficient in predicting gazing behavior when the visual field contains social information (e.g., Nyström and Holmqvist, 2008; Birmingham et al., 2009a,b; Fletcher-Watson et al., 2009; Zwickel and Vo, 2010; Hall et al., 2011; Scheller et al., 2012; Suda and Kitazawa, 2015).

Moreover, the present study revealed important insights into the time course of the influences of social attention and low-level saliency. Previous research indicated that saliency-based attention models perform best for the very first fixations after stimulus onset (e.g., Parkhurst et al., 2002; but see Tatler et al., 2005). Additionally, several studies yielded evidence that a bias of directing gaze to other people may be present (e.g., Birmingham et al., 2008a, 2009b; Fletcher-Watson et al., 2008) or perhaps even strongest (e.g., Fletcher-Watson et al., 2008; Kano and Tomonaga, 2011; Xu et al., 2014; Wang et al., 2015) for such early fixations. Furthermore, there is support that such tendencies may not be solely explained by physical saliency (e.g., Birmingham et al., 2009b). The current study provides a more comprehensive distinction of both influences on early eye movements by means of detailed analyses of the first five fixated scene locations in complex naturalistic social and non-social scenes. Importantly, we not only replicated the finding that low-level saliency is higher at locations fixated very early as compared to locations looked at later, but we also showed both the preference for social features (especially heads) as well as the lower correspondence between fixations and physical saliency for scenes including social elements as compared to scenes depicting solely non-social content to be most pronounced for the very first fixations. These findings indicate that social attention and physical saliency interact in predicting the very first fixations during scene processing. More specifically, they demonstrate that the preferential processing of social features in complex naturalistic scenes does not only rely on a volitionally controlled mechanism but instead reflects the influence of a reflexive and automatic process that trades off physical saliency by the presence of social features on very early fixations. This conclusion substantiates prior reports which yielded support that certain high-level visual stimuli (e.g., faces) may partly capture attention automatically (e.g., Cerf et al., 2009; Kano and Tomonaga, 2011) and further corroborates previous studies providing evidence that social features may have the potential to override low-level saliency (e.g., Nyström and Holmqvist, 2008; Birmingham et al., 2009b). Building onto our findings, future research should try to reveal further characteristics of the automatic versus controlled mechanisms underlying the allocation of attention in complex naturalistic scenes.

One may speculate why the magnitude of the reduced correspondence between fixations and physical saliency for scenes containing people already had its maximum at the first fixation whereas the preferential processing of social features (especially heads) only reached its peak at the second fixation. A possible explanation may be that although attention was driven reflexively by social elements instead of low-level saliency beginning from the very first fixation, observers’ gaze often reached these social features not until the second fixation. This explanation is supported by the fact that the average of the mean distance of social features to the scene center (*M* = 9.60°, *SD* = 3.41° for heads; *M* = 9.71°, *SD* = 3.16° for bodies), i.e., where participants were instructed to look at before exploring a scene, was about twice as large as the average saccade lengths (*M* = 4.67°, *SD* = 0.66° for social scenes; *M* = 4.86°, *SD* = 0.73° for non-social scenes).

In addition, the results of the currently applied modeling approach revealed further interesting aspects of the role of social influences in the context of saliency-based attention models. Several previous modeling studies provided evidence that considering other sources of information (e.g., locations of faces) besides low-level saliency may improve the power of predicting fixations (e.g., Cerf et al., 2008, 2009; Marat et al., 2013; Xu et al., 2014; Parks et al., 2015; for a review, see Tatler et al., 2011). Furthermore, a number of studies yielded support that such other sources of information may even have a higher predictive value than physical saliency (e.g., Coutrot and Guyader, 2014; Xu et al., 2014). However, previous studies mostly only relied on the approach of comparing models encompassing diverse sets of predictive features. Thus, research which allows for disentangling different attentional influences in one and the same model is very sparse (see e.g., Vincent et al., 2009; Coutrot and Guyader, 2014). Moreover, with just a few exceptions (e.g., Wang et al., 2015), previous studies almost completely neglected to investigate the interplay of considered predictors. Importantly, the present study particularly targeted both aspects by applying a recently proposed linear mixed model analysis approach (Nuthmann and Einhäuser, 2015). This approach enabled us to demonstrate explicitly that social features, physical saliency, their interaction, and a central bias are significant predictors of gazing behavior within a shared model. Notably, explicitly considering central bias as a predictor within the modeling approach gave us the possibility to examine the influences of physical saliency and social features above this general spatial viewing tendency. In accordance with their extraordinary attentional prioritization, heads of depicted people constituted the strongest of all predictors. Completed with evidence from our model comparisons, our findings show that social features add substantially to the prediction of fixations beyond low-level saliency. Interestingly, the interaction terms revealed that a scene location was not only looked at more when it generally included heads or bodies but additionally when these social features were physically salient at the same time. This finding, which is in line with supplementary analyses of a recent study that did not consider interaction terms as model predictors (Wang et al., 2015), demonstrates the influences of physical saliency and social features to be not totally independent from each other. Thus, the current approach indicates that future models of human visual attention need to integrate social influences and low-level saliency as well as their interaction.

In the context of the present study, it seems interesting that previous research yielded evidence that a number of mental disorders which are particularly characterized by deficits in the context of social situations (e.g., autism spectrum disorders, social phobia) could be associated with altered orienting toward socially relevant information (e.g., Pelphrey et al., 2002; Horley et al., 2003; Birmingham et al., 2011; Boll et al., 2016). However, just as research on social attention in general, prior studies investigating patients with mental disorders mostly relied on isolated face stimuli (e.g., Pelphrey et al., 2002; Horley et al., 2003; Boll et al., 2016) and only very sparsely on more naturalistic visual material (e.g., Fletcher-Watson et al., 2009; Birmingham et al., 2011; Freeth et al., 2011). In addition, although recent studies on (social) information processing in clinical populations began to consider saliency-based attention models (e.g., Fletcher-Watson et al., 2009; Freeth et al., 2011; Wang et al., 2015), research of both domains is not connected sufficiently. Importantly, the current study makes a substantial contribution for closing the very same gap regarding attentional processes in healthy human beings. Thus, we also demonstrate aspects which might be highly relevant for future studies aiming at enhancing the understanding of (altered) social attention in people with mental disorders such as autism or social phobia (cf., Itti, 2015). In particular, our application of Nuthmann and Einhäuser’s (2015) linear mixed model approach could be adapted to contrast clinical and healthy control groups regarding the power of social features, physical saliency, their interaction, and other potentially interesting factors in guiding attention.

Despite its strengths, some limitations of the current study are also worth mentioning. First, when comparing different sets of stimuli (e.g., social and non-social scenes), one has to bear in mind that the sets could potentially differ in other than the intended dimensions. However, we carefully applied the same selection criteria (e.g., complexity, depth of field) to all scenes to minimize the risk of potential confounds and demonstrated both scene types to have comparable distributions of low-level image saliency and to be similar both in image complexity and in emotional quality. In addition, comparing human observers’ gazing behavior between social and non-social scenes is in fact more a strength than a weakness of the current study because previous research has so far completely neglected to make this important comparison. Second, it has to be taken into account that we examined eye movements of humans watching visual scenes under one specific viewing condition: free exploration. Although free viewing is frequently considered to basically resemble some kind of natural viewing mode (e.g., Parkhurst et al., 2002; but see Tatler et al., 2011), several studies yielded support that different task instructions (e.g., free viewing vs. visual search) have a substantial impact on observers’ gazing behavior (e.g., Yarbus, 1967; Welchman and Harris, 2003; Birmingham et al., 2007, 2008a; Foulsham and Underwood, 2007; Rothkopf et al., 2007; Einhäuser et al., 2008a; Fletcher-Watson et al., 2008, 2009). Therefore, future research should try to examine whether the findings of the present study hold true under varying task demands. Third, recent research provided evidence that social context (e.g., sitting with others in a waiting room or eating together with them) may modulate the extent to which humans attend to other people (e.g., Laidlaw et al., 2011; Foulsham et al., 2011; Wu et al., 2013; Gregory et al., 2015). Although the current study made important advancements in terms of ecologically valid stimulus material, our laboratory experiment did not allow for estimating the influence of social context. Thus, it will be an important challenge for future research to test the findings from laboratory experiments in various social contexts (see also e.g., Kingstone, 2009).

In sum, the present study makes a distinct contribution to elucidate the role of human social attention in the context of saliency-based attention models. We showed that observers strongly prefer social features (especially heads) over physically salient locations during the exploration of naturalistic visual scenes. Importantly, our findings indicate that this prioritization of social information does not only rely on a volitionally controlled mechanism but especially on a reflexive and automatic process. The current study strongly challenges the generalizability of saliency-based attention models and demonstrates the importance of considering social influences when investigating the driving factors of human visual attention.

## Author Contributions

AE and MG designed the study. AE collected and analyzed the data. MG supervised data analysis. AE and MG wrote and reviewed the manuscript.

## Conflict of Interest Statement

The authors declare that the research was conducted in the absence of any commercial or financial relationships that could be construed as a potential conflict of interest.

## Footnotes

1 https://www.r-project.org

2 Please note that even though GBVS was implemented in the current study, all of our major results were qualitatively similar both with the traditional Itti and Koch model (Itti et al., 1998; Itti and Koch, 2000) and with a very recent model (Boolean Map Saliency; Zhang and Sclaroff, 2016) which achieved high performance on the MIT Saliency Benchmark website (Bylinskii et al., 2016).
